# Lymphatic insufficiency leads to distinct myocardial infarct content assessed by magnetic resonance T_RAFFn_, T_1ρ_ and T_2_ relaxation times

**DOI:** 10.1038/s41598-023-28219-6

**Published:** 2023-01-28

**Authors:** Elias Ylä-Herttuala, Taina Vuorio, Sanna Kettunen, Svetlana Laidinen, Seppo Ylä-Herttuala, Timo Liimatainen

**Affiliations:** 1 A.I. Virtanen Institute for Molecular Sciences, University of Eastern Finland, Kuopio, Finland; 2 Clinical Imaging Center, Kuopio University Hospital, Kuopio, Finland; 3 Heart Center and Gene Therapy Unit, Kuopio University Hospital, Kuopio, Finland; 4 Research Unit of Medical Imaging, Physics and Technology, University of Oulu, P.O. Box 5000, 90014 Oulu, Finland; 5 Department of Diagnostic Radiology, Oulu University Hospital, Oulu, Finland

**Keywords:** Cardiovascular diseases, Cardiology, Cardiovascular biology, Experimental models of disease, Preclinical research, Diagnostic markers, Biological models, Imaging

## Abstract

The role of cardiac lymphatics in the pathogenesis of myocardial infarction (MI) is unclear. Lymphatic system regulates cardiac physiological processes such as edema and tissue fluid balance, which affect MI pathogenesis. Recently, MI and fibrosis have been assessed using endogenous contrast in magnetic resonance imaging (MRI) based on the relaxation along a fictitious field with rank n (RAFFn). We extended the RAFFn applications to evaluate the effects of lymphatic insufficiency on MI with comparison to longitudinal rotating frame (T_1ρ_) and T_2_ relaxation times. MI was induced in transgenic (TG) mice expressing soluble decoy VEGF receptor 3 that reduces lymphatic vessel formation and their wild-type (WT) control littermates for comparison. The RAFFn relaxation times with rank 2 (T_RAFF2_), and rank 4 (T_RAFF4_), T_1ρ_ and T_2_ were acquired at time points 0, 3, 7, 21 and 42 days after the MI at 9.4 T. Infarct sizes were determined based on T_RAFF2_, T_RAFF4_, T_1ρ_ and T_2_ relaxation time maps. The area of differences (AOD) was calculated based on the MI areas determined on T_2_ and T_RAFF2_, T_RAFF4_ or T_1ρ_ relaxation time maps. Hematoxylin–eosin and Sirius red stained histology sections were prepared to confirm MI locations and sizes. MI was detected as increased T_RAFF2_, T_RAFF4_, T_1ρ_ and T_2_ relaxation times. Infarct sizes were similar on all relaxation time maps during the experimental period. Significantly larger AOD values were found together with increased AOD values in the TG group compared to the WT group. Histology confirmed these findings. The lymphatic deficiency was found to increase cardiac edema in MI. The combination of T_RAFF2_ (or T_RAFF4_) and T_2_ characterizes MI and edema in the myocardium in both lymphatic insufficiency and normal mice without any contrast agents.

## Introduction

The lymphatic system collects extracellular fluids from the tissue having a role in several pathophysiological processes, which have been recently revisited^1^. The lymphatic system regulates many processes involved in cardiac physiology and pathology, such as inflammatory reactions caused by cell death^2^, tissue fluid balance^3^, reverse cholesterol transport^4^ and atherosclerosis^5,6^, which can eventually change the function of the myocardium when tissue homeostasis is disturbed. The role of lymphatic vessels in myocardial infarction (MI) is currently understudied^8–10^ and this role in the pathogenesis of MI still remains unclear but is probably more important than previously thought^11–13^. It has also been hypothesized that an impaired lymphatic system could evoke edema, which can lead to an imbalance of nutrients in the damaged tissue, furthermore, affecting cardiac output^12^.

MRI has been widely used in the detection of acute reversible injury, the extent of edema and chronic injury^14–16^. Elevated T_2_ relaxation time and tissue extracellular water content display a linear relationship in MI^14,17^. T_2_ relaxation time assesses edema through increased extracellular water content^14,17^ and can differentiate acute and chronic MI based on edema^18^. The acute MI includes a mixture of reversible edema and irreversible MI. Reversible edema leads to an overestimation of the MI size in the T_2_ based imaging and relaxation time mappings^19,20^.

Currently the gold standard to detect chronic irreversible injury using MRI is contrast agent (CA) based late gadolinium enhancement (LGE). Gadolinium (Gd) chelates may cause acute adverse and chronic reactions^15–21^. The endogenous contrasts, such as the longitudinal rotating frame (T_1ρ_), are beneficial in avoiding side effects of Gd^21^ and possibly give more information about the heterogeneity of the myocardial damage area^22^. Additionally, the injection of Gd through the tail vein injection route is not always possible in preclinical rodent setups, which limits especially follow-up imaging.

In T_1ρ_, the magnetization relaxes during on-resonance radiofrequency (RF) irradiation^16–20,24^. The increase of T_1ρ_ has been related to the formation of granulation tissue and fibrosis after MI^16–20,24^. The area with elevated T_1ρ_ corresponds to the fibrotic MI area verified by LGE in both mice^23,24^ and in humans^25^. However, T_1ρ_ measurement leads to a relatively high specific absorption rate (SAR), which is a limiting factor in clinical applications.

One method to reduce SAR of rotating frame relaxation is relaxation along a fictitious field (RAFF) with rank n (RAFFn)^26,27^. RAFFn method is produced by nested sine amplitude and cosine frequency modulated RF pulses operated in a sub-adiabatic regime producing a fictitious magnetic field component^26–28^. Fictitious field increases the spin-locking field strength without increasing the SAR^26,27^. The RAFFn is sensitive to molecular motions that are on the same frequency as its spin-locking field. These molecular motions may change in granulated, fibrotic and scar tissues compared to intact myocardium^26,27^. RAFFn has shown its potential in preclinical cardiac studies including fibrotic MI, where it was increased at the fibrotic area of MI in both acute and chronic phase without use of CAs^23^. The extent of MI area determined based on RAFFn was shown to correlate highly to MI area from the histology and LGE^23^. Results suggest that the RAFFn is sensitive at revealing the biological events that occur in both acute and chronic phases of MI, such as cell death, inflammation, granulation tissue formation and fibrosis, making it a suitable candidate for determining the effects of insufficient lymphatic system after the MI^23^. The RAFFn was able to also detect the presence of fibrosis in hypertrophic cardiomyopathy^29^.

Here, we studied the effects of insufficient cardiac lymphatic system on RAFFn relaxation times (T_RAFF2_, T_RAFF4_), T_1ρ_ and T_2_ relaxation times during the development of MI. As a reference, left ventricular (LV) functional parameters were measured with MRI and infarct sizes were confirmed by histology.

## Materials and methods

### Animal model

Mice expressing the soluble decoy VEGF receptor 3 (sVEGFR3) in LDLR/ApoB background that reduces lymphatic vessel formation in the heart and elsewhere in the body formed a transgenic group (TG). For a control group, wild-type (WT) littermates were selected^6^. Permanent MI was induced by ligating the left anterior descending (LAD) coronary artery in 16 TG and 23 WT mice^30,31^. Briefly, mice were anesthetized with inhalation of isoflurane (Univentor-400, Univentor, Zejtun, Malta) 4% induction before the LAD operation with anesthesia being maintained with 2% during the operation. The heart was exposed and pushed out from the thorax and LAD was ligated approximately at midline level of the heart using a 6–0 silk suture (Mansfield, MA, USA). After the LAD ligation, the heart was placed back into its original location. After the surgery, the mice received buprenorphine (0.3 mg/ml) (Temgesic (RB Pharmaceuticals, Slough, UK)) 0.05–0.1 mg/kg and carprofen (50 mg/ml) (Rimadyl (Pfizer Oy Animal Health, Helsinki, Finland)) 5 mg/kg for analgesia subcutaneously. Analgesia was repeated on the first and the second day after the surgery. All animal experiments were performed according to ARRIVE guidelines together with guidelines and protocols approved by the University’s Institutional Animal Care and Finnish National Animal Experimental Board for the use and care of laboratory animals and the animal experiments were carried out in accordance with the Act on the Protection of Animals Used for Scientific or Educational Purposes (497/2013).

### MRI

Mice were imaged before the MI operation (day 0) and 3 days (n = 11 in TG group, n = 14 in WT group), 7 days (n = 8 in TG group, n = 9 in WT group), 21 days (n = 8 in TG group, n = 11 in WT group) and 42 days (n = 1 in TG group, n = 4 in WT group) after the MI. The number of animals varied between time points due to mortality. The MRI experiments were carried out using a horizontal 9.4 T magnet (Varian Inc. Palo Alto, CA, USA) controlled by a Bruker Biospec console (Bruker GmbH, Ettlingen, Germany). A quadrature volume transceiver coil with inner diameter of 35 mm (Rapid Biomed GmbH, Ettlingen, Germany) was used. Mice were anesthetized for MRI experiments with 4% isoflurane mixed with oxygen and nitrogen gases in a ratio of 1:3. The isoflurane level was reduced to 1–1.5% during the imaging. Mouse body temperature was kept close to a natural temperature level (37 C°) by a circulating warm water pad placed under the mouse. ECG was measured using needle electrodes from the fore paws and respiration was controlled by a pneumatic pillow placed under the mouse abdomen. Both signals were registered using Model 1025 (Small Animal Instruments Inc., NY, USA) and they were used to gate all MRI experiments.

Multi-slice short-axis ECG triggered and respiration gated view cine images covering the whole heart were acquired using gradient echo based fast imaging with steady state precession (FISP) readout sequence. The imaging parameters for cine images were field of view (FOV) = 4 × 4 cm^2^, slice thickness = 1 mm, matrix size = 192 × 192, echo time (TE) = 1.9 ms, repetition time (TR) = 8.0 ms, scan TR = 99.0 ms, flip angle = 10° with the number of frames were 10–11 depending on the mouse’s heart rate. Depending on the size of the heart, 8–12 slices were imaged.

The RAFFn relaxation times (T_RAFFn_) were acquired applying two preparation modules RAFF2 or RAFF4^26,27^ pulses waveforms (nominal RF power (γB_1_/(2π)) 1250 Hz and 648 Hz, respectively, and duration 1.13 ms^27^). Pulses were added to pulse trains with durations of 0, 9.1, 18.1 and 36.2 ms resulting four differently weighted images separately for both RAFF2 and RAFF4. Despite the pulse train duration, acquisition occurred in the same cardiac phase.

The T_1ρ_ data was acquired using a rotating frame preparation module consisting of an adiabatic half-passage (AHP) pulse (power = 1250 Hz, duration = 3.0 ms), continuous wave spin-lock-pulse (power = 625 Hz) with spin-lock time (TSL) of 0.4, 9.4, 27.4 and 45.4 ms resulted four differently weighted images. After spin-lock pulse, an AHP-back pulse (power 1250 Hz, duration 2.0 ms) was applied^23^. Readout occurred at the same cardiac phase for all weighted images.

The T_2_ measurements contained Hahn double echo preparation which included an AHP excitation-pulse (power = 1250 Hz, duration = 3.0 ms), two hyperbolic secant 1 (HS1)-pulses^32^ (power = 1250 Hz, duration = 4.5 ms) and an AHP-back pulse (power = 1250 Hz, duration = 3.0 ms). Symmetric delays were applied between the pulses, resulting in total TEs of 0.05, 2.3, 4.5, and 14.0 ms, which resulted four differently weighted images. Delays in front of the T_2_ preparation were also added similarly as in RAFFn and T_1ρ_.

B_1_ was measured by applying a hard pulse with power of 625 Hz and pulse durations 0, 0.25, 0.5, 0.75, 1.0, 1.25, 1.5 and 1.75 ms^33^.

The FISP-readout sequence was used to acquire a single short-axis slice with the same geometry at the mid-ventricular level for all relaxation time and B_1_ measurements with the following parameters: FOV = 4 × 4 cm^2^, slice thickness = 1 mm, matrix size = 256 × 256 (for B_1_ measurements matrix size was 128 × 128), TE = 1.9 ms, TR = 14.9 ms, scan was dependent on both heart and respiratory rate. The minimum delay between weighting pulses was chosen as 1460 ms to make imaging time faster while signal loss from steady state was still reasonable.

### Histology

Hearts were perfused with phosphate buffered saline through the LV and then immersed in sucrose containing 4% paraformaldehyde. After 4–16 h, the liquid was changed to 15% sucrose. The hearts were then paraffin-embedded and 4 μm thick cross-sections of the heart were stained with hematoxylin eosin (HE) and Sirius red (SR) to distinguish the fibrotic and collagen area of the infarcted myocardium^8^. Histological sections were photographed with a light microscope (Nikon Eclipse, Ni-E, Tokyo, Japan). Histology was done at day 21 and at day 42 after the LAD ligation.

### Data-analysis

All MRI images were analyzed using the Aedes software (http://aedes.uef.fi/) in Matlab (Mathworks Inc. Natick, MA, USA). Relaxation time (T_RAFF2_, T_RAFF4_, T_1ρ_ and T_2_) and B_1_ maps were reconstructed from signal intensities pixel-by-pixel manner using the Aedes software. All maps were fitted using linear function into logarithms of signal intensities. Regions of interest (ROI), which included the MI and remote areas, were manually traced with visual delineations of MI and remote areas. An overlay ROI was used to ensure that the enhanced relaxation times were specific from the myocardium area. The overlay ROI was formed based on raw weighted MR image. Additionally, cine and histology images were used to localize the fibrotic MI area on the relaxation time maps. End systolic volume, end diastolic volume, ejection fraction (EF), and stroke volume were defined based on the endocardial border evident in cine images^23^.

Infarct percentage analysis was done by manually traced midline length-based method with a function of (L_(infarct)_/L_(circumference)_) × 100%, where L_(infarct)_ denotes measured arc length of MI area from either T_RAFF2_, T_RAFF4_, T_1ρ_, T_2_, SR or HE stained section and L_(circumference)_ denotes the midline length of the whole myocardium^34^. The area of difference (AOD) with respect to the T_2_-defined MI area (A_2_), which include both the infarct and edema regions, was calculated with a function of ((A_2_ – A)/A) × 100%, where A denotes the area of infarct size without edema in either T_RAFF2_, T_RAFF4_, or T_1ρ_ relaxation time map^35^. Additionally, the edema area of the LV outside the exact infarct area in SR stained sections were calculated with open source ImageJ (National Institutes of Health, MA, USA) software by calculating the extracellular space between myocytes inside the whole left myocardium. After this calculation, AOD was calculated with similar way as MRI data. Co-registration between the MRI and the histological section was based on visual agreement.

### Statistics

All numerical values are given as mean ± standard error of mean (SEM). Two-way ANOVA with Bonferroni post hoc test for multiple comparisons was applied to compare the spatial and temporal changes between the infarct and remote areas of myocardium. The Pearson’s correlation was calculated to evaluate the association between MRI relaxation time maps and the histology sections. Analyses were performed using GraphPad Prism software (GraphPad Software, La Jolla, CA, USA).

### Ethics approval and consent to participate

All surgical procedures were performed according to protocols approved by the Finnish Committee for the use and care of laboratory animals.

## Results

**Figure 1:**
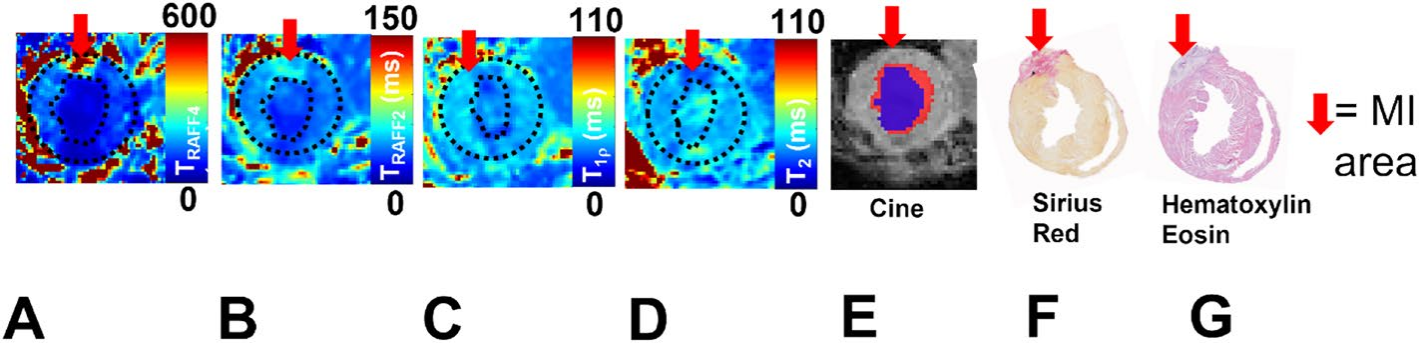
Relaxation time maps T_RAFF2_, T_RAFF4_, T_1ρ_ and T_2_ (**A**–**D**), respectively, cine (**E**)- and a corresponding Sirius red (**F**) and hematoxylin eosin (**G**) stained section from infarcted TG mouse heart at the last (21 day) time point after the MI. Co-registration between different weightings are done by using the same geometry in each image. Dotted black lines on the relaxation time maps delineate the location of the myocardium, which are based on weighted images and overlayed on the relaxation time maps. Red arrows indicate the infarct area. The blue area in the cine image indicates the left ventricle area in systole and blue and red areas together are the diastole phase of the heart cycle.

**Figure 2:**
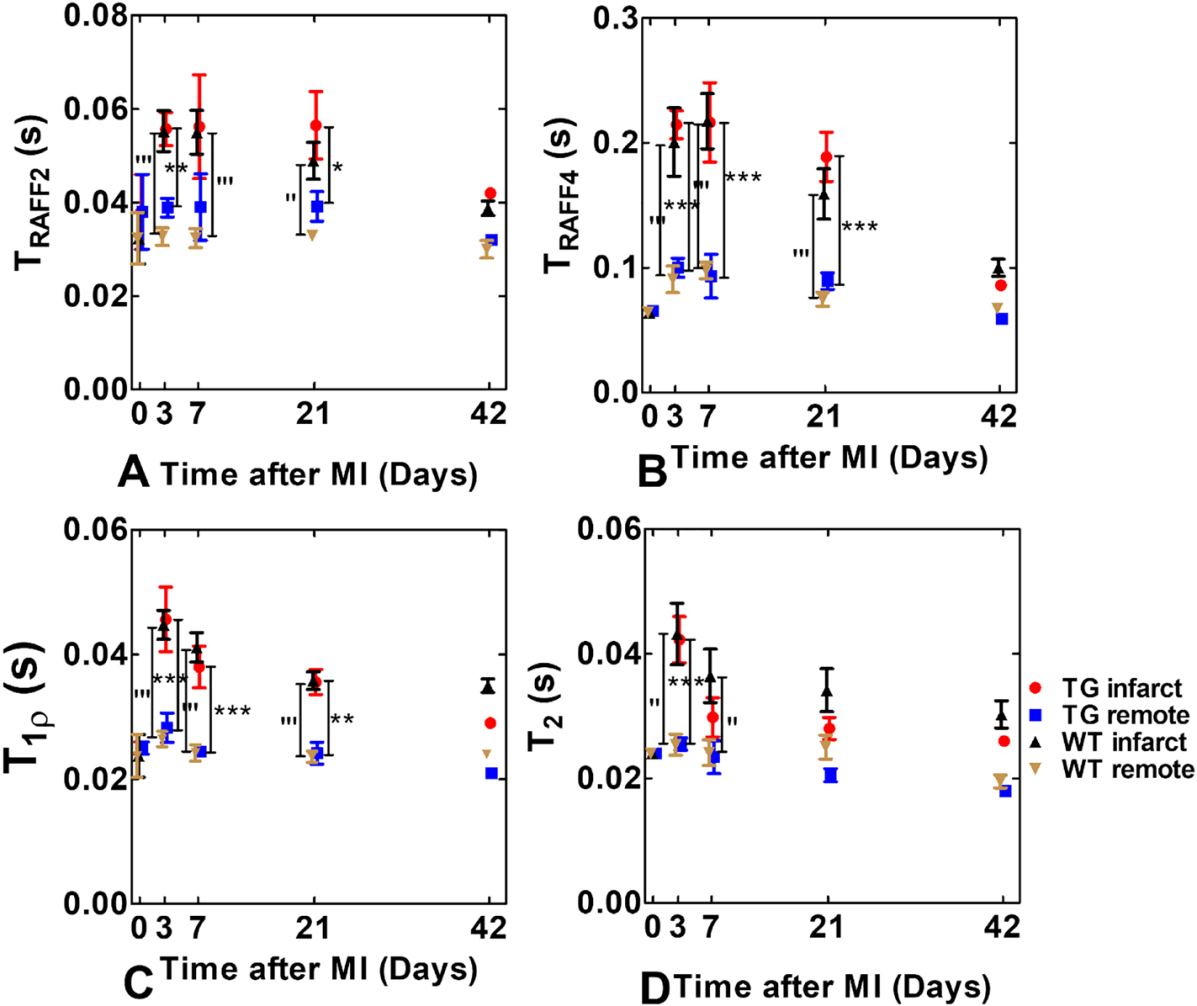
Relaxation times for TG and WT mice as a function of time. Rotating frame relaxation times (**A**–**C**) and T_2_ relaxation time (**D**). Values represent mean ± SEM. *P < 0.05, **P < 0.01, ***P < 0.001 for significant differences between TG infarct and remote areas calculated by Two-Way ANOVA with Bonferroni post hoc test for multiple comparisons. Similarly, ’P < 0.05, ’’P < 0.01, ’’’P < 0.001 for significant differences between WT infarct and remote areas calculated by Two-Way ANOVA with Bonferroni post hoc test for multiple comparisons.

The MI area was visible in all relaxation time maps as increased relaxation times (T_RAFF2_, T_RAFF4_, T_1ρ_ and T_2_). Areas with increased relaxation times were co-located with an akinetic area in cine images and the fibrous area in SR-stained sections (Fig. 1). All relaxation times increased significantly in the damaged MI area as compared to remote areas in both TG and WT groups (Fig. 2). Similar relaxation times were detected in MI areas in both groups (Fig. 2). The B_1_ field varied between 570 and 660 Hz from nominal 625 Hz in the myocardium including all time points.

**Figure 3:**
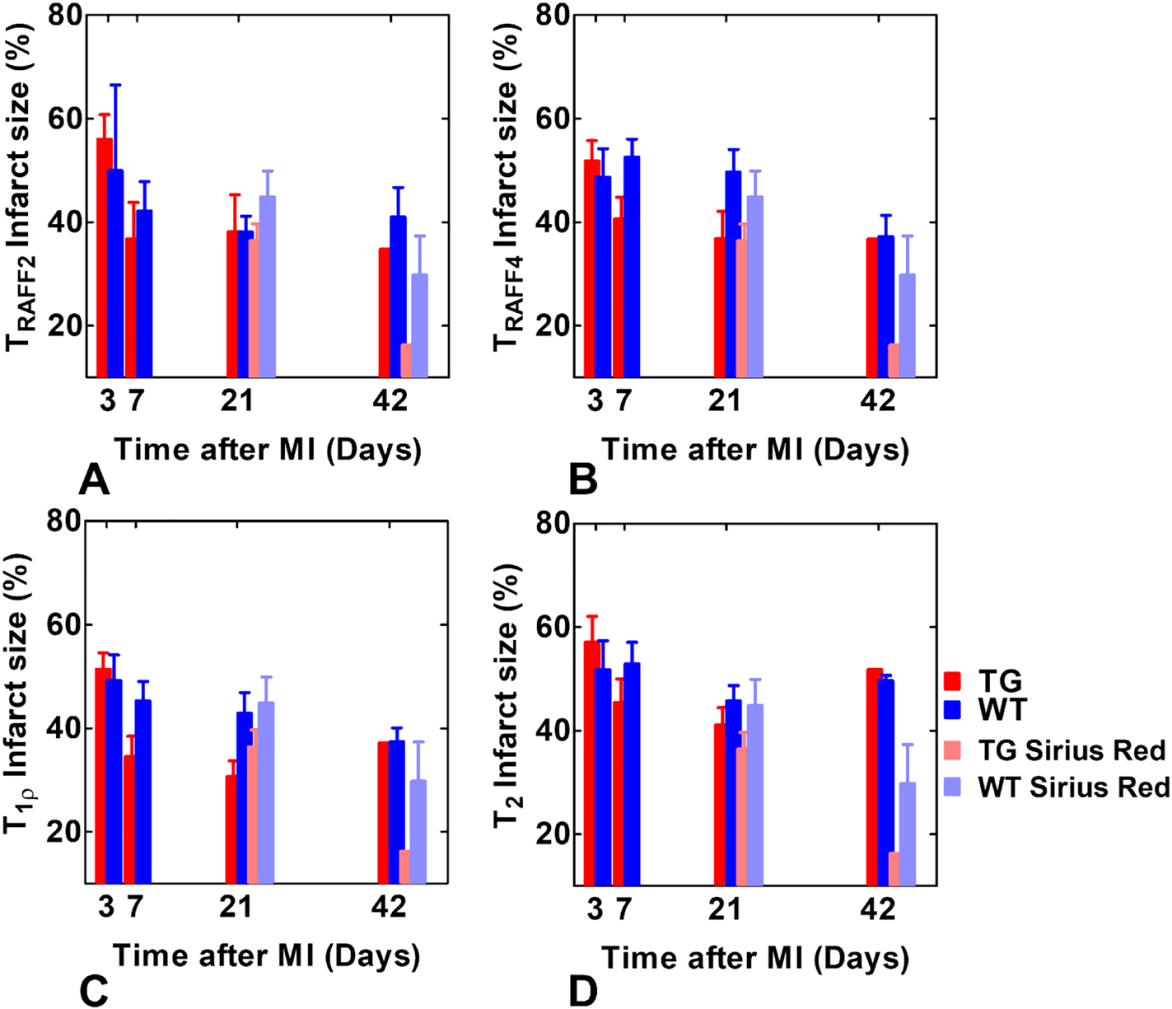
Infarct percentage sizes based on relaxation time maps T_RAFF2_ (**A**), T_RAFF4_ (**B**), T_1ρ_ (**C**) and T_2_ (**D**) at time points of 3, 7 and 21 days after MI (mean ± sem (%)). The same calculations were done for infarct percentage size determined in Sirius red stained histology sections (bars at days 21 and 42 time points).

Infarct percentage sizes measured on T_RAFF2_, T_RAFF4_, T_1ρ_ and T_2_ relaxation time maps were in the similar sizes as a function of time and the infarct percentage sizes determined with SR-stained histology sections were similar as determined based on MRI relaxation time maps at 21 days after the MI (Fig. 3). However, infarct percentage sizes determined with SR-stained histology sections at 42 days after the MI were smaller compared to the infarct percentage sizes determined by different relaxation time maps (Fig. 3). The highest correlations between the fibrotic infarct percentage size based on the SR-stained histology and the infarct percentage size based on the relaxation times were found with T_RAFF4_ (R^2^: 0.81, *P* < 0.05) and T_RAFF2_ (R^2^: 0.67, *P* = 0.09) (Table 1).

**Table 1:** Correlations between infarct percentage size as determined by the Sirius red stained histology and infarct size based on relaxation times at 21 days after MI (*P < 0.05, ^P = 0.09).

Mice group	T_RAFF2_	T_RAFF4_	T_1ρ_	T_2_
TG	0.56	0.81*	0.51	0.21
WT	0.67^	0.57	0.46	0.20

**Figure 4:**
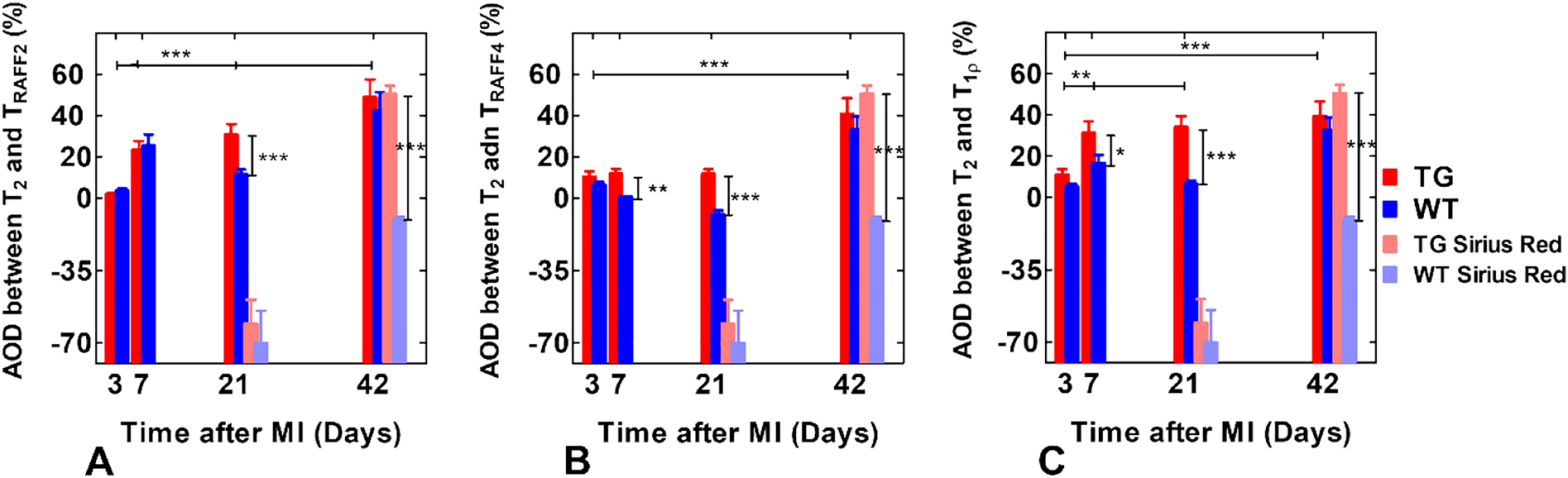
Area of difference (AOD) was determined by subtracting infarct areas based on T_RAFF2_ (**A**), T_RAFF4_ (**B**) and T_1ρ_ (**C**) from the area based on T_2_ at every time point after MI (mean ± sem (%)). The same calculations were done by subtracting exact infarct area from edema area in Sirius red stained histology sections (bars at days 21 and 42 time points). Statistical significance is calculated by Two-Way ANOVA with Bonferroni post hoc test for multiple comparisons (*P < 0.05, **P < 0.01, ***P < 0.001).

**Figure 5:**
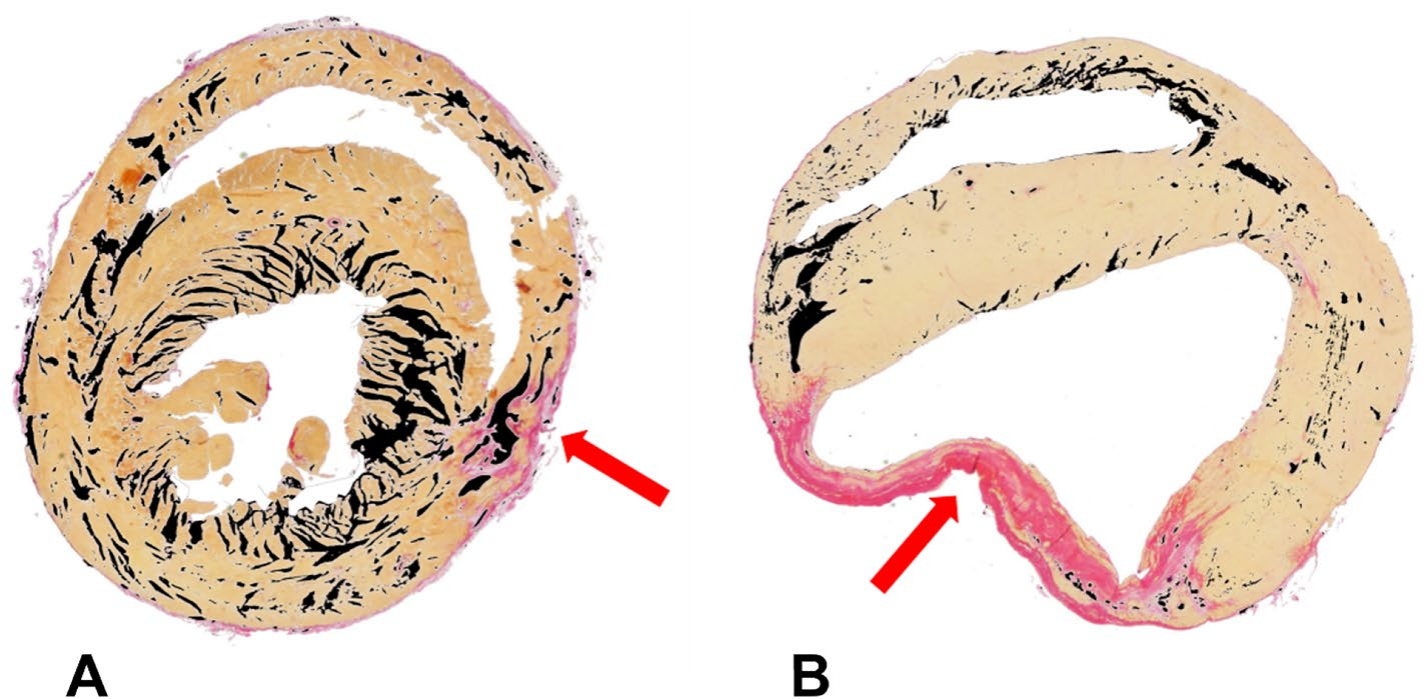
An example images from Sirius red stained histology sections from TG (**A**) and WT (**B**) mice at 21 days after MI. Black areas in the myocardium indicates the extracellular space, measured by digitally thresholding the empty white space within the myocardium. Red arrows indicate the infarct area.

The difference between the MI area determined based on the T_2_ map and the rotating frame relaxation time maps denoted as AOD were significantly larger in the TG group compared to the WT group at days 7 and 21 after the MI (Fig. 4). AOD values in TG group were increased significantly on T_RAFF2_, T_RAFF4_ and T_1ρ_ relaxation time maps as a function of time (Fig. 4). Also, SR-stained histology sections revealed large AOD values in the TG group compared to the WT group at both 21 and 42 days after the MI (Figs. 4 and 5).

**Figure 6:**
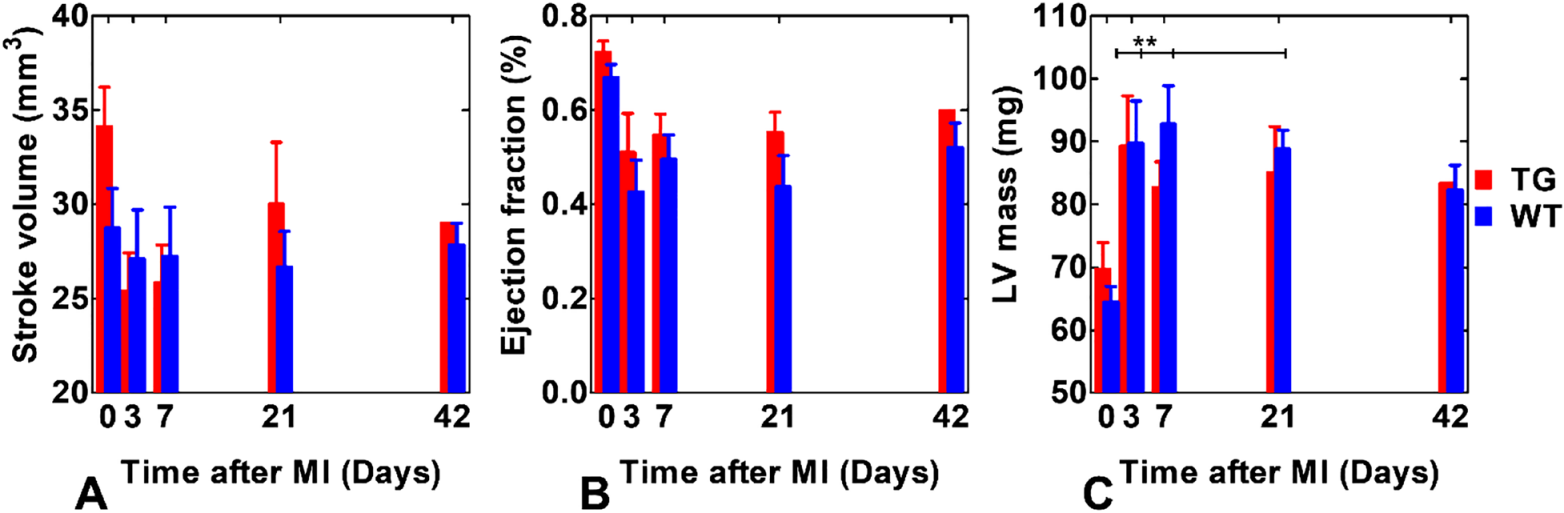
Cardiac functionality and functional parameters of TG and WT mice at every time point for stroke volume (**A**), ejection fraction (**B**) and LV mass (**C**). Statistical significance is calculated by Two-Way ANOVA with Bonferroni post hoc test for multiple comparisons (**P < 0.01).

Cardiac functional parameters were similar between the groups, however, WT group had slightly higher, but non-significant, values compared to TG group (Tables 2 and 3). The stroke volume and EF were similar between the groups during the study period (Fig. 6A,B). LV mass increased significantly in both groups after MI (Fig. 6C).

**Table 2:** Cardiac functional parameters based on cine images in TG mice (mean ± sem (%)), where EDV is end-diastolic volume and ESV is end-systolic volume.

	EDV [mm^3^]	ESV [mm^3^]	INFARCT SIZE [%]
DAY 0	47.5 ± 3.6	13.4 ± 1.9	0
DAY 3	59.2 ± 14.5	33.7 ± 14.5	41.6 ± 0.1
DAY 7	46.9 ± 4.7	21.0 ± 2.6	35.9 ± 0.4
DAY 21	54.9 ± 6.2	24.8 ± 4.5	41.0 ± 0.7
DAY 42	48.4 ± 0	19.4 ± 0	6.7 ± 0.3

**Table 3:** Cardiac functional parameters based on cine images in WT mice (mean ± sem (%)), where EDV is end-diastolic volume and ESV is end-systolic volume.

	EDV [mm^3^]	ESV [mm^3^]	INFARCT SIZE [%]
DAY 0	43.5 ± 4.5	14.8 ± 2.4	0
DAY 3	69.0 ± 6.7	41.9 ± 8.2	67.3 ± 0.4
DAY 7	60.1 ± 8.0	32.9 ± 7.7	38.8 ± 0.7
DAY 21	66.4 ± 10.9	39.5 ± 11.3	39.7 ± 0.2
DAY 42	53.8 ± 3.3	26.0 ± 4.2	26.5 ± 3.4

## Discussion

The T_RAFF2_, T_RAFF4_, T_1ρ_ and T_2_ relaxation times were mapped to study the effects of cardiac lymphatic system on the fibrotic MI area at various time points. We determined infarct percentage sizes in the TG mice with an insufficient lymphatic system and their littermates at various time points after the MI based on increased relaxation times in the myocardium. As a reference, LV functional parameters were measured with MRI, and the infarct percentage sizes and AOD values were confirmed by histology staining.

The normal lymphatic system maintains the fluid balance in healthy myocardium. The impaired lymphatic system in TG group was presumed to lead to more extensive fluid accumulation in TG compared to WT group. Larger AODs in the TG than in the WT group from both MRI at later time points and histology supports this presumption. Interesting finding was that edema area was larger and infarct sizes from the SR-stained histological sections were smaller at day 42 compared to day 21 after the MI. This might be due to cardiac remodeling, which occurred between those time points. In a previous mouse MI study^8^, a high correlation was found between increased T_RAFF2_ and T_RAFF4_ maps and histology-derived infarct area, which is in line with the current results and might indicate that this is the case also in the presence of edema. Therefore, the combination of T_2_ and rotating frame relaxation time maps, either T_RAFF2_ or T_RAFF4_, may provide valuable information about the edema and irreversible MI area without contrast agents.

In VEGFR3 mouse model, lymphatic system is impaired with altered cardiac lymphatic vessel morphology and decreased lymphangiogenesis^8^. In the same study, cardiac lymphatic vessels formed a dense network at the border zone of the MI area in the WT group resulting in a sharp MI area in the histology sections compared to a more diffuse MI area in the TG mice^8^. This was supported by our findings that the TG group had a diffuse MI area compared to the WT group representing fibrosis formation in histology sections at later time points. The result indicates that the TG mice had increased cell death, edema, granulation tissue formation and fibrotic tissue at the MI border zone, which is reflected as increased AOD values. Also, the LV mass increased in both groups after MI, which is in line with current knowledge^36^. Based on the cine images, cardiac functional parameters were similar in both groups indicating that the lymphatic impaired mouse heart can still compensate MI related challenges. However, there is a decrease in infarct size between 21 and 42 days after MI in both groups based on cine images, which might be due to successful cardiac remodeling and adaptation to the changed biological circumstances.

Rotating frame relaxations including T_1ρ_ and T_RAFFn_ are selectively sensitive for molecular processes occurring at the effective field frequency, which is a combination of RF pulse, possible off-resonance from Larmor frequency and fictitious field, while T_2_ is non-selectively sensitive for low frequency fluctuations. All measured relaxation times were similar in the MI area between the TG and WT groups indicating that relaxation times in the MI are independent of the lymphatic vessels in this mouse model. Progressions of T_RAFF2_, T_RAFF4_, T_1ρ_ and T_2_ were found similar after MI in both groups. The cardiac remodeling occurred and may explain the decreased relaxation times. The RAFFn methodology presented in this study has been modified for clinical use, where promising results have been found in the chronic MI^37^.

Cardiac lymphatic flow has been studied in humans^38^ and large animals^39^ after MI showing that MI decreases cardiac lymph flow. Decreased lymphatic flow leads to edema^40^ having an impact on adverse remodeling of myocardium. However, our findings were not able to confirm this adverse remodeling of the myocardium. Additionally, lymphangiogenic therapy has been shown to improve cardiac function after MI in mice^40^ as well as resolving cardiac edema and fibrosis in rats^41^, which further supports the role of the lymphatic system in MI recovery.

As limitations of the study, number of animals in both groups was low especially at the latest time point. However, the number of animals is still adequate to draw conclusions. One limitation was also the lack of LGE data to determine scar in chronic MI in MRI. Partial volume effect needs to be considered since large MI leads to thinner myocardium and can potentially cause the signal leakage from LV blood to the MI area. Additionally, manual tracing of the ROIs may add bias to MI areas and average relaxation times.

As a conclusion, the lymphatic deficiency was found to increase cardiac edema in MI. The combination of T_RAFF2_ (or T_RAFF4_) and T_2_ can characterize MI and edema in the myocardium in both lymphatic insufficiency and normal mice without any contrast agents.

## Data Availability

The datasets used and/or analyzed during the current study are available from the corresponding author on reasonable request.
